# Quantitative and longitudinal monitoring of cancer cell invasion in a three-dimensional *in vitro* model of oral cancer using optical coherence tomography

**DOI:** 10.1038/s41598-025-28471-y

**Published:** 2025-11-27

**Authors:** Kenta Haga, Yoshifumi Kamimura, Manabu Yamazaki, Akinori Funayama, Yuko Saito, Masako Kida, Jun-ichi Tanuma, Kenji Izumi

**Affiliations:** 1 Division of Biomimetics, Faculty of Dentistry & Niigata University Graduate School of Medical and Dental Sciences, Niigata, 951-8514 Japan; 2 Division of Reconstructive Surgery for Oral and Maxillofacial Region, Faculty of Dentistry & Niigata University Graduate School of Medical and Dental Sciences, Niigata, 951-8514 Japan; 3 Division of Oral Pathology, Faculty of Dentistry & Niigata University Graduate School of Medical and Dental Sciences, Niigata, 951-8514 Japan; 4 SCREEN Holdings Co., Ltd., Kyoto, 602-8585 Japan; 5 Division of Oral and Maxillofacial surgery, Faculty of Dentistry & Niigata University Graduate School of Medical and Dental Sciences, Niigata, 951- 8514 Japan; 6 Division of Biomimetics, Faculty of Dentistry & Niigata University Graduate School of Medical and Dental Sciences, 2-5274 Gakkocho-dori, Chuo-ku, Niigata, 951-8514 Japan

**Keywords:** Optical coherence tomography, Deep learning, Oral squamous cell carcinoma, Cancer cell invasion, 3D organotypic model, Cancer-associated fibroblasts, Tumor microenvironment, Cancer, Oncology

## Abstract

We previously developed a three-dimensional (3D) organotypic culture model of oral squamous cell carcinoma (OSCC) by incorporating cancer-associated fibroblasts to replicate oral tissue architecture and the tumor microenvironment (TME). This model provides a relevant platform for investigating cancer cell invasion. Optical coherence tomography (OCT), a noninvasive, high-resolution imaging technique, enables both real-time and longitudinal observations. This study assessed the applicability and feasibility of combining OCT with deep learning for the quantitative, longitudinal monitoring of cancer cell invasion in our 3D model. OCT effectively captured cross-sectional images and identified three regions—original cancer cell region, invasive cancer cell region, and stromal layer—based on scattering intensity and optical density, demonstrating nondestructive visualization of tissue microarchitecture. Sequential OCT imaging facilitated 3D image reconstruction and repeated monitoring. Planimetric and volumetric analyses of 3D OCT images revealed internal structural alterations and enabled comparative evaluation of invasion behaviors across OSCC cell types, TME conditions, and culture durations. Moreover, the invasiveness parameter obtained from 3D OCT images strongly correlated with histomorphometric-based data, confirming its reliability. These findings support the use of OCT imaging as a promising tool for noninvasive, quantitative assessment of invasiveness in organotypic cancer models.

**Supplementary Information:**

The online version contains supplementary material available at 10.1038/s41598-025-28471-y.

## Introduction

Optical coherence tomography (OCT), specifically spectral domain OCT (SD-OCT) in this study, is a label-free, nondestructive imaging technique that provides high-resolution, cross-sectional, and real-time 3D images of biological structures comparable to that obtained with histopathology^1^. OCT detects backscattered and reflected light from tissue layers based on low-coherence interferometry. Variations in tissue optical properties generate signals from different depths^2^. OCT is widely used for diagnosing diseases in organs such as the eye^3^. Initially developed for ophthalmology, its use has expanded to nontransparent tissues such as the skin and oral mucosa. Although optical scattering limits imaging depth, recent advancements allow high-resolution imaging up to 2 mm^4^.

In oncology, OCT is particularly well-suited for screening, guided biopsy, intraoperative imaging, and monitoring tumor response because of its label-free, depth-resolved imaging capabilities. Cancer tissues, which exhibit disorganized architecture and altered scattering properties, are distinguishable from normal tissues *in vivo*. Clinically, OCT has been employed to image cancers in various organs, including the breast, brain, bladder, gastrointestinal tract, respiratory tract, reproductive tracts, skin, and oral cavity^5^. Furthermore, the high soft-tissue contrast of OCT—resulting from differences in refractive index—enhances early cancer diagnosis^5–8^.

Two-dimensional (2D) cell culture in *in vitro* cancer research lacks the complexity of the tumor microenvironment (TME) due to the absence of microarchitectures, extracellular signals, and physiological conditions^9,10^. In contrast, 3D cultures—such as scaffold-free spheroids and organoids—better mimic *in vivo* environments, providing more accurate insight into cell–cell and cell–matrix interactions, as well as therapeutic responses^11^. These 3D models have transformed research by closely simulating human tissues and organ behavior, providing a deeper understanding of human disease. In addition to its clinical applications in cancer detection and early diagnosis, OCT offers appropriate spatiotemporal resolution for the nondestructive investigation of complex oncological cellular dynamics* in vitro*, providing cross-sectional imaging of internal tissue microstructures, micron-scale resolution for cellular-level imaging, and rejection of multiply scattered light^5,6,12^. These properties enable imaging depths of 2–3 mm, optimal for assessing subsurface structures in 3D tissue cultures^13^. This facilitates longitudinal monitoring of size, structure, viability, and cell death within the same sample over time^12,14–16^.

Previously, we developed a scaffold-based 3D oral cancer model using organotypic culture, composed of oral cancer cells and underlying stroma containing cancer-associated fibroblasts^17^. This model replicates the TME and is appropriate for histological and molecular studies of cancer cell invasion^17–19^. OCT can visualize subsurface structures and their interactions with scaffold matrixes, making it a powerful tool for analyzing such 3D models^12,13^. Therefore, OCT can be applied to the 3D organotypic oral cancer model as a valuable platform for quantitative, longitudinal monitoring of cancer invasion. This study aimed to evaluate the applicability and feasibility of OCT imaging to noninvasively and reproducibly characterize the biological structure of a 3D model and oral cancer cell invasion. Using 3D spatial information reconstructed from OCT images, we proposed two image-derived parameters: the planimetric surface area of the “hypothetical invasion front (HIF)” and the volumetric measurement of the “invasive cancer cell region (ICCR).” Four types of 3D models were constructed using HSC-2 and HSC-3 oral squamous carcinoma cells along with cancer-associated fibroblasts (CAFs) and NOFs as stromal cells. These models were scanned via OCT at Days 14 and 21 of culture during their development. To assess the applicability and feasibility of OCT imaging, we presented specific qualitative and quantitative imaging results by comparing cancer cell invasion behaviors across three factors: OSCC cell type, tumor microenvironment (TME), and manufacturing period, represented by two oral cancer cells, two stromal cells, and two culture durations, respectively. These findings highlight the potential of OCT for studying invasive cancer cell behavior *in vitro*.

## Materials and methods

### Ethical approval

Primary human oral fibroblasts (NOFs) were harvested from normal oral mucosa tissue. The protocol for harvesting human oral mucosa tissue samples was approved by the Niigata University Ethical Committee (Approval: #2015–5018) under the following title: “Translational research toward advanced regenerative medicine of oral mucosa: From bench to bedside.” All patients received sufficient information about the study, and all participating individuals signed an informed consent form. All experiments were performed in accordance with the relevant guidelines and regulations.

### Cells and culture methods

HSC-2 (RRID: CVCL_1287) and HSC-3 (RRID: CVCL_1288), both human oral squamous cell carcinoma (OSCC) cell lines derived from oral cancer, were obtained from the Riken BRC Cell Bank (Tsukuba, Ibaraki, Japan). HSC-2 cells, representing well-differentiated OSCC, and HSC-3 cells, representing poorly differentiated OSCC, were originally established from a metastatic lymph node of a floor-of-the-mouth cancer and a tongue cancer, respectively^20^. Both OSCC cell lines were authenticated by short tandem repeat profiling in May 2020, and all experiments were conducted using mycoplasma-free cells. CAFs derived from non-small cell lung cancer were obtained from Cellular Engineering Technologies, Inc. (Coralville, IA, USA). Primary normal oral fibroblasts (NOFs) were established using an explant culture technique and serially cultured as previously described^17,21^.

All cells were maintained in Dulbecco’s modified Eagle’s medium (DMEM; Thermo Fisher Scientific, Waltham, MA, USA), supplemented with 10% fetal bovine serum (FBS; Thermo Fisher Scientific), gentamicin (5.0 µg/mL), and amphotericin B (0.375 µg/mL; Thermo Fisher Scientific) in a humidified atmosphere of 5% CO_2_ and 95% air at 37 °C. CAFs and NOFs at passages 3–5 were used in the experiments.

### Fabrication of 3D organotypic oral cancer models

Four types of 3D cancer models, comprising an oral cancer layer (HSC-2 or HSC-3 cells) and an underlying stromal layer (CAFs or NOFs), were constructed as described previously^17^. Initially, the stromal layer was prepared. Three milliliters of type I collagen solution (Nitta Gelatin, Osaka, Japan), containing 5.0 × 10^5^ cells at a density of 1.67 × 10^5^ cells/mL, was plated into a six-well tissue culture insert (Greiner Bio, Roskilde, Denmark) and incubated under a submerged condition at 37 °C in DMEM supplemented with 10% FBS in a humidified atmosphere of 5% CO_2_ and 95% air. An acellular collagen solution was plated as a control condition. After 2 days, the solidified stromal layer was detached from the walls of the tissue culture insert using a 200-µL micropipette tip, inducing stromal layer contraction. After 5 days, 50 µL of HSC-2 or HSC-3 cell suspension, containing 5.0 × 10^5^ cells in DMEM with 10% FBS, was seeded onto the top surface of the stromal layer (Day 7 from the start). The composite of OSCC cells and the stromal layer was cultured under submerged conditions for an additional 7 days. Depending on the stromal components, three types of 3D models were developed: the 3D cancer-CAF model, 3D cancer-NOF model, and 3D cancer-alone model. On Day 14, the 3D oral cancer models were raised to an air–liquid interface and cultured for another 7 days. By Day 21, the fabrication of the 3D cancer models was complete. The models were fixed in 4% paraformaldehyde. Paraffin-embedded sections were prepared for histological analysis using hematoxylin-eosin (HE) staining. Some 3D cancer models were also fixed on Day 14 to examine oral cancer cell invasion.

### OCT scanning protocol of 3D oral cancer models and imaging workflow

**Fig. 1:**
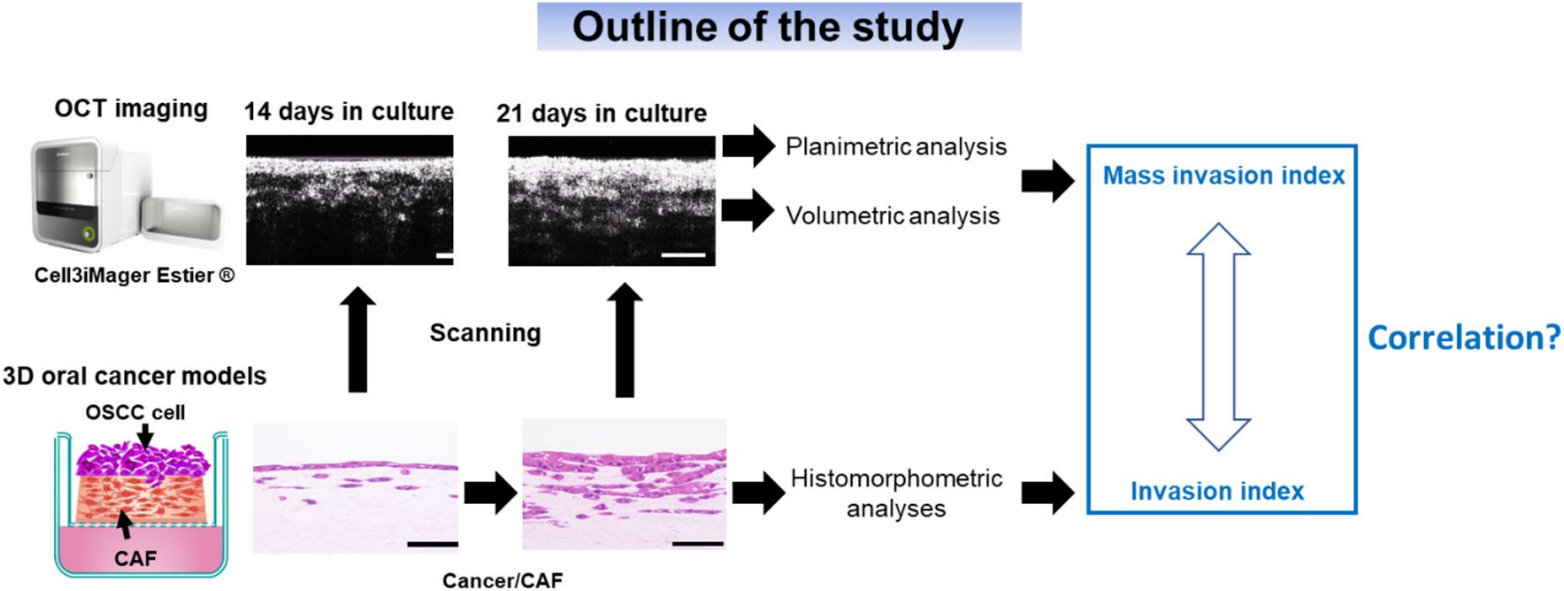
Research scheme of the study. Schematic of the research plan, methodology, and overall study design.

Quantitative and longitudinal monitoring of HSC-2 and HSC-3 cell invasion in 3D cancer models was performed using the SD-OCT system Cell iMager Estier (SCREEN Holdings, Kyoto, Japan), which enables label-free and noninvasive 3D live imaging. The research scheme for this study is illustrated in Fig. 1. OCT imaging was performed as described previously^22^. Briefly, the system used a superluminescent diode (SLD) light source with a center wavelength of 890 nm (bandwidth = 790–1020 nm, N.A. = 0.3, beam diameter = 10 μm, output = 10 mW). The SLD output is coupled into a single-mode optical fiber and split into the 3D cancer model and reference arms via a fiber coupler. Reflections from both arms are recombined at the coupler and detected by the spectrometer. To ensure that the optical path lengths of the 3D cancer model and the reference arm are matched, the optical path length of each component is adjusted based on a refractive index of 1.37. The optical resolution in the XY plane is 3 μm, and the axial (Z-direction) resolution is 0.86 μm.

For OCT imaging, the 3D cancer model was transferred into a 60-mm culture dish (Corning, New York, NY, USA) using forceps and positioned inverted before scanning. One milliliter of culture medium was added to the dish to maintain moisture during imaging. These procedures ensured clear OCT imaging without light source attenuation. At least three locations were scanned during each session. The positions of each 3D cancer model were marked with Indian ink to ensure consistent imaging locations. OCT images of the 3D cancer models were captured at 1-µm intervals along the Z-axis, from the cancer cell layer to the underlying the stromal layer, using a high magnification lens. This imaging process required approximately 20 min. After placing the dish, a region of interest (ROI) (500 μm × 500 μm) was selected at the center of the 3D cancer model. Cross-sectional images (X–Z) of the approximate ROI were acquired to ensure consistency of the scanning location over time. On Days 14 and 21 of the fabrication process, OCT imaging was conducted on the same 3D cancer models, enabling longitudinal monitoring of approximately the same ROI in each model and ensuring imaging reproducibility. The ROI in the OCT images was identified using the Indian ink markings and used for subsequent histological examination.

### Quantitative and longitudinal monitoring of cancer cell invasion using 2D and 3D parameters based on 3D reconstructed OCT images

To eliminate noise from the culture medium and the dish surrounding the 3D cancer models, the original OCT images (X–Y scan area = 500 μm × 500 μm; Z-axis scan range = 300–600 μm) were processed using Cell Visualizer software (SCREEN Holdings), and the data were reconstructed using isotropic voxels with dimensions of 1 μm × 1 μm × 1 μm.

In the cross-sectional OCT images, OSCC cells (HSC-2 and HSC-3 cells), stromal cells (CAFs and NOFs), and the collagen matrix were represented in 16-bit gray-scale. For quantitative monitoring of oral cancer cell invasion within these 3D cancer models, cells were segmented as distinct objects using semantic segmentation based on a convolutional neural network, a primary deep learning algorithm used in image recognition^23–26^. Target cells were detected by extracting segmented cells from other surrounding structures. During the deep learning process, OSCC cells and stromal cells were labeled on cross-sectional images using HE-stained sections of the 3D models. We used these labeled cross-sectional images as training data.

Using a deep learning–based semantic segmentation algorithm, OSCC cells and stromal cells were binarized on the cross-sectional images. The experimental procedure was adapted from a previously described method with minor modifications^27,28^. The training dataset comprised approximately 55 labeled images—500 pixels on the X-axis and 300–600 pixels on the Z-axis—for the construction of the deep learning–based model. In this process, cancer cells within both the original and ICCRs were labeled as training data based on HE-stained sections. For quantitative analysis of cancer cell invasion into the stromal layer, two parameters, planimetric and volumetric, were proposed based on the reconstructed 3D images: the surface area of the HIF and the total volume of the ICCR, respectively. First, the total volume of the entire cancer cell region was measured by counting voxels using the “3D object counter” function in ImageJ (National Institutes of Health, Bethesda, MD, USA). The total volume of the ICCR, which represents the volumetric invasion parameter, was calculated by subtracting the volume of the original cancer cell region. This measurement quantifies the volume of invasive cancer cell clusters or islands that are spatially separated from the original cancer cell mass. Next, the surface area of ​​the HIF, representing the planimetric parameter, was generated and quantified. The HIF was defined as the plane formed by regions of high signal intensity contiguous with the original cancer cell region. This plane was assumed to be convoluted and located within the ICCR, as the invasive cancer clusters or islands (indicated in red) were detached from the original cancer cell region. Finally, the mass invasion index was calculated using the following formula: [1 − (total volume of the ICCR / total volume of the entire cancer region)], which corresponds to the two-dimensional invasion index.

### Measurement of the depth of invasion and invasion index in 3D organotypic cancer models based on histological examination

We quantitatively assessed cancer cell invasion in both 3D cancer models by measuring “the depth of invasion” and the “invasion index,” as described previously^17^. Briefly, five random fields were selected under high-power magnification (×200) within a single HE-stained section. Four model types, comprising two cancer cell lines and two stromal cell types (HSC-2/CAFs, HSC-2/NOFs, HSC-3/CAFs, and HSC-3/NOFs), were examined at two time points (Days 14 and 21 of culture), resulting in eight distinct manufacturing conditions. Based on histological observations (Supplementary Fig. 1A and B), the 3D organotypic oral cancer models were divided into three regions from top to bottom: (1) the original cancer cell region, (2) the ICCR, and (3) and the stromal layer. To measure the “depth of invasion” and “invasion index,” the following parameters were evaluated using ImageJ. The “(1) original cancer cell region” was defined as the area primarily composed of cancer cells initially seeded on the stromal layer and located on the model surface, without infiltration into the underlying “(3) stromal layer” (Supplementary Fig. 1A and B). The invasion front was identified as the boundary between the infiltrating cancer cells and the surrounding stromal layer (Supplementary Fig. 1B). The “(2) ICCR” was defined as the area between the original cancer cell region and the portion of cancer cells infiltrating the stromal layer (Supplementary Fig. 1B). Accordingly, the “depth of invasion” was defined as the maximum vertical distance from the bottom plane of the original cancer cell region to the invasion front (Supplementary Fig. 1B). The “invasion index” was calculated as 1 − (area of original cancer cell region [mm^2^]/ area of total cancer cell region [mm^2^]), where a value of “0” indicates no infiltration of cancer cells into the stromal layer (the cancer cell layer remains entirely above the stromal layer) and a value of “1” indicates complete infiltration (the stromal layer is fully exposed to the air surface)^17,29^.

### Statistical analysis

Data are presented as mean ± standard deviation (S.D.). To compare the 3D cancer models comprising HSC-2 and HSC-3 OSCC cells with those comprising CAFs and NOFs as stromal cells, differences in mean values were analyzed using Welch’s *t*-test. To compare models analyzed on Days 14 and 21 during manufacturing, differences in mean values were assessed using the Mann–Whitney *U* test. A p-value of < 0.05 was considered statistically significant. All statistical analyses were performed using EZR (Jichi Medical University, Tochigi, Japan), a graphical user interface for R (The R Foundation for Statistical Computing, Vienna, Austria).

## Results

### Visualization of cancer cell invasion in 3D oral cancer models via OCT imaging

**Fig. 2:**
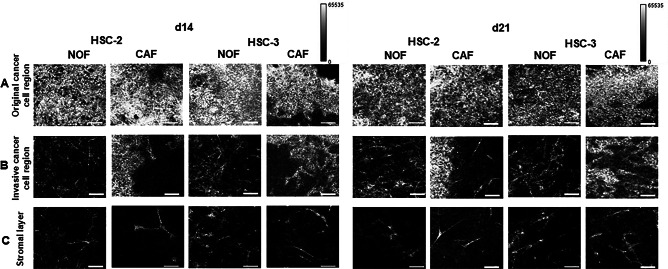
Representative OCT images (X–Y slices) at three levels from top to bottom within 3D oral cancer models under eight conditions (HSC-2 and HSC-3 cells; repopulated with NOFs and CAFs; 14 and 21 days in culture). (**A**) X–Y slice of the original cancer cell region. Scale bars 100 μm. (**B**) X–Y slice of the invasive cancer cell region. Scale bar: 100 μm. (**C**) X–Y slice of the stromal layer. Scale bar: 100 μm. A color bar shown by gray-scale indicates the signal intensity (0 to 65535) of this OCT images.

First, we applied OCT imaging to the 3D cancer models cultured for 14 and 21 days to determine whether infiltrating cancer cells within the underlying stromal layer could be visualized and whether three regions could be distinguished (Fig. 1). OCT images acquired along the Z-axis (X–Y slice) revealed strong signal intensity from cellular components, producing a bright granular area on the top surface (Fig. 2A). Moreover, cancer cell clusters or islands appeared heterogeneous in the OCT images (Fig. 2B). In contrast, the extracellular matrix exhibited weak signal intensity, resulting in dark regions, although spindle-shaped fibroblastic cells could be visualized within this layer (Fig. 2C). The characteristics of OCT images were consistent across different levels within each layer: the original cancer cell region, invasive cancer cell region, and stromal layer (Supplementary Fig. 2A–C). The resolution of the OCT images was sufficient to visualize microarchitecture features such as infiltrating cancer cell clusters/islands and fibroblasts.

**Fig. 3:**
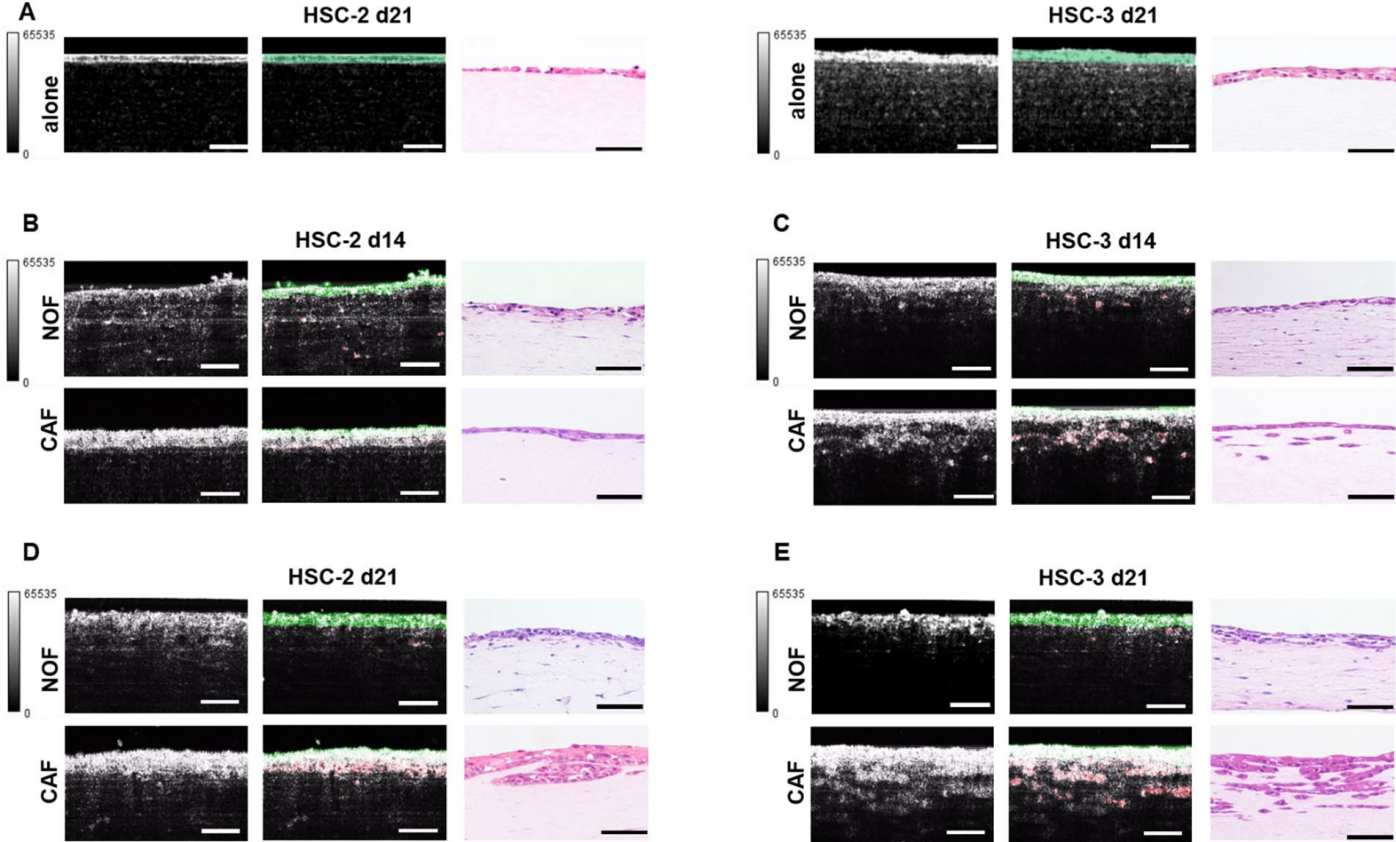
Representative panels of OCT cross-sectional (X–Z) images (left), deep learning–processed images (middle), and histological images (right) of 3D oral cancer models under 10 conditions (HSC-2 and HSC-3 cells; acellular stromal layer or repopulated with NOFs and CAFs; and 14 and 21 days in culture). (**A**) Representative panel of 3D oral cancer models in which HSC-2 or HSC-3 cells were seeded on top an acellular stromal layer cultured for 21 days. White scale bar: 100 μm; black scale bar: 50 μm. (**B**) Representative panel of 3D HSC-2 oral cancer models in which NOFs or CAFs repopulated the stromal layer cultured for 14 days. White scale bar: 100 μm; black scale bar: 50 μm. (**C**) Representative panel of 3D HSC-3 oral cancer models in which NOFs or CAFs repopulated the stromal layer cultured for 14 days. White scale bar: 100 μm; black scale bar: 50 μm. (**D**) Representative panel of 3D HSC-2 oral cancer models in which NOFs or CAFs repopulated the stromal layer cultured for 21 days. White scale bar: 100 μm; black scale bar: 50 μm. (**E**) Representative panel of 3D HSC-3 oral cancer models in which NOFs or CAFs repopulated the stromal layer cultured for 21 days. White scale bar: 100 μm; black scale bar: 50 μm. Cohesive invasion patterns and well-defined invasion fronts were observed in the HSC-2 CAF models, whereas the invasive cancer cell region in HSC-3 CAF models was more prominent and larger than in the HSC-2 CAF models.

Second, we examined cross-sectional OCT images (X–Z slices) within the approximate ROI to assess depth-resolved visualization of cancer cell invasion across the eight conditions of the 3D cancer models (Fig. 3). A continuous granular layer of strong signal intensity at the top, corresponding to the original cancer cell region, thickened with increasing culture duration across all models. Below this bright granular layer, areas of heterogeneously gray or irregularly strong signal intensity were observed, which were identified as the ICCR. The ICCR was clearly identified in the HSC-3 cancer model images (Fig. 3C,E). An area with no strong signal intensity was observed at the base of the model, representing the stromal layer. Similar to the X–Y slices, the cross-sectional OCT images showed patterns corresponding to the three regions along the Z-axis, whereas the 3D cancer model without repopulating CAFs/NOFs exhibited only two regions (Fig. 3B–E vs. Fig. 3A). Accordingly, OCT imaging appeared to differentiate the three regions within the 3D cancer models in a manner broadly consistent with the corresponding histological findings (Fig. 3B–E vs. Supplementary Fig. 1A and B). Notably, the 3D cancer-CAF models with HSC-3 cells exhibited a granular and heterogeneous pattern and a larger ICCR over time, more pronounced than in other models. This facilitated clear depth-resolved OCT visualization and enabled the monitoring of cancer cell invasion over time. Moderately reflective regions were occasionally observed within the ICCR, likely resulting from reflection interference caused by strong adjacent signals from cancer cell clusters or islands.

**Fig. 4:**
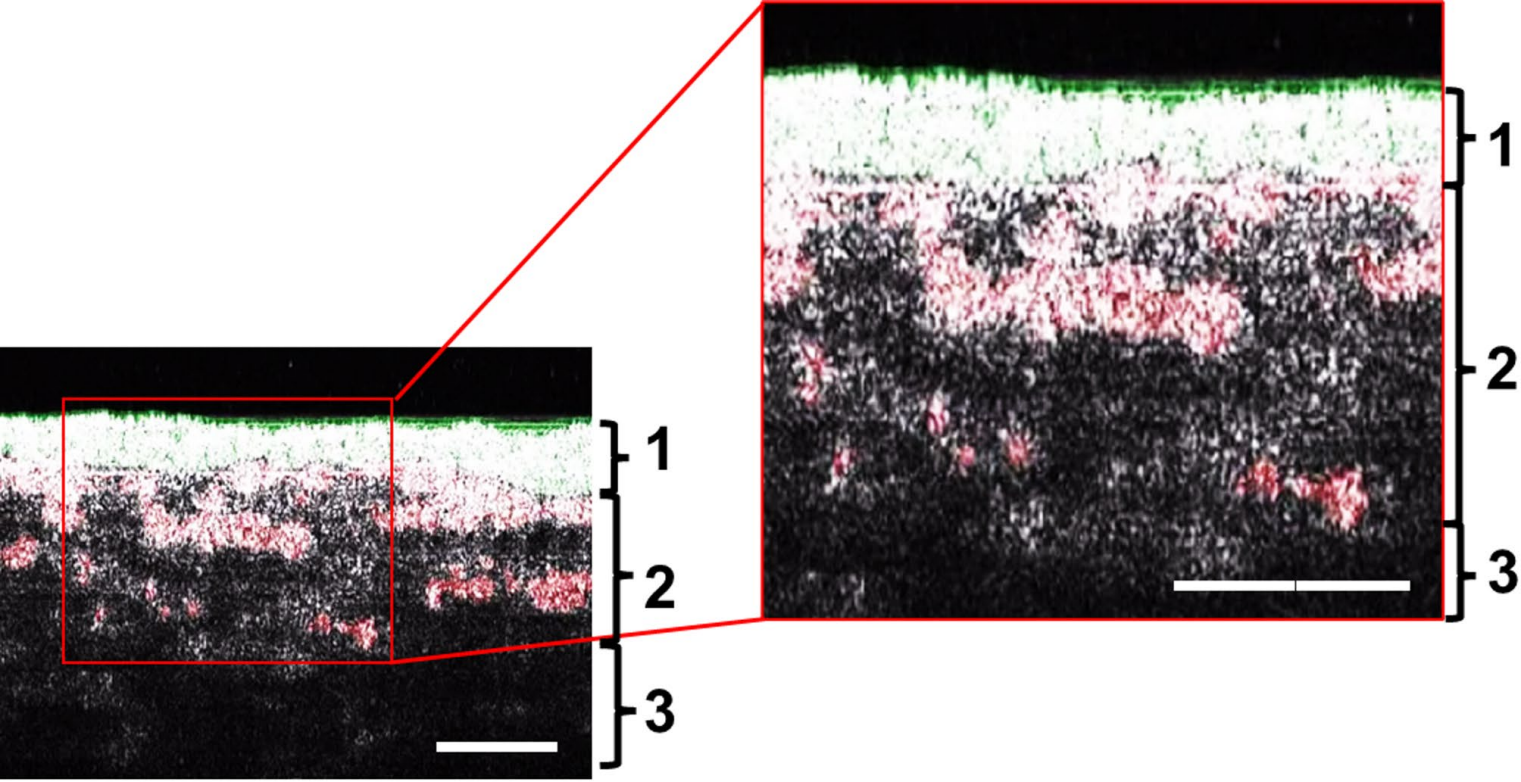
OCT cross-sectional image (X-Z slice) of the HSC-3 CAF model at 21 days in culture after deep learning processing. The right panel shows a higher-magnification view of the boxed area outlined with red solid line in the left panel. This image demonstrates the resolution of the OCT system used in this study. Brackets 1, 2, and 3 indicate the original cancer cell region, invasive cancer cell region, and stromal layer, respectively.

Finally, we applied deep learning–based segmentation to the original OCT images, allowing the original cancer cell region and the ICCR to be labeled in green and red, respectively (Figs. 3A–E and 4; Supplementary Movies 1 and 2). To address signal attenuation in OCT imaging—which varies with imaging depth and tissue properties—we employed a deep learning–based approach rather than relying solely on conventional threshold-based binarization. This method enables more robust detection of cancer cell regions by incorporating signal intensity and spatial context, thereby mitigating depth-related bias and improving segmentation accuracy. The microstructures colored red occupied a larger area in the 3D cancer-CAF models with HSC-3 cells than in other models and expanded over time, consistent with histological observations. Furthermore, HSC-2 cells exhibited a cohesive invasion pattern, whereas HSC-3 cells exhibited a more island-like invasion pattern.

### Histological findings and invasive behavior of 3D oral cancer models

In addition to OCT imaging, histological and histomophometric examinations were performed (Fig. 1). HSC-2 and HSC-3 cells infiltrated the underlying stromal layer, exhibiting distinct invasion patterns, consistent with our previous findings^17^. In the 3D HSC-2 cancer models, the original cancer cell region was thicker in the CAF model than in the NOF model, suggesting a greater proliferation capacity of cancer cells when co-cultured with CAFs (Fig. 3B,D). A cohesive invasion pattern and a well-defined invasion front were observed. In contrast, 3D HSC-3 cancer models exhibited finger-like and island-type invasion (Fig. 3C,E). This invasive behavior was more pronounced in co-culture with CAFs and after 21 days in culture, as evidenced by a thicker ICCR and a less distinct invasion front than HSC-2 cells. Conversely, in models co-cultured with NOFs or with collagen matrix alone, infiltrating cancer cells were rarely observed (Fig. 3A,B,D).

**Fig. 5:**
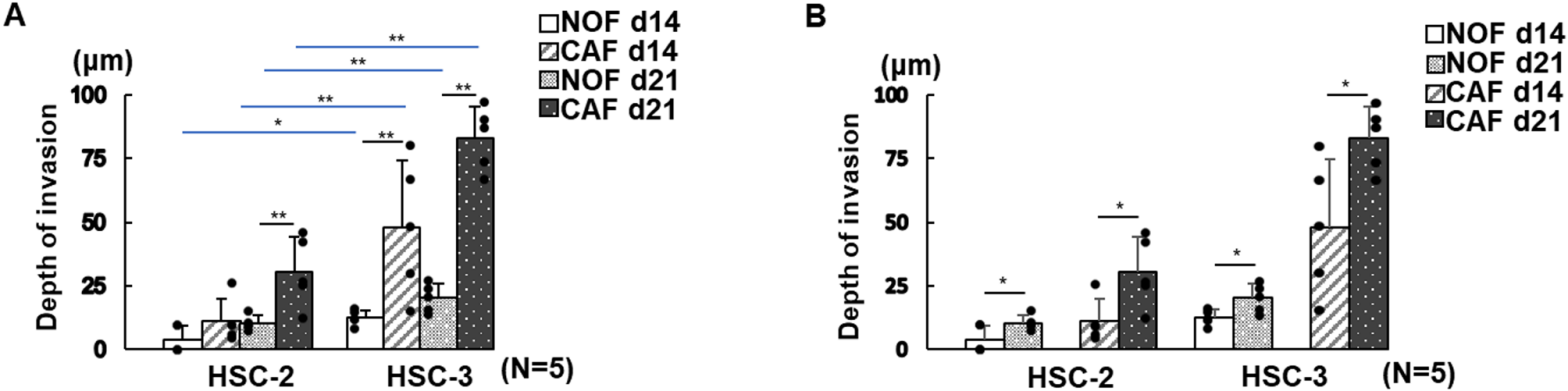
Depth of invasion in 3D oral cancer models under different conditions. (**A**) Quantification of the “Depth of invasion (µm)” comparing four independent conditions (HSC-2 vs. HSC-3 and CAFs vs. NOFs). Data are presented as means ± S.D. ns: not significant, *p < 0.05, **p < 0.01. (*N* = 5). (**B**) “Depth of invasion (µm)” comparing models cultured for 14 vs. 21 days. Data are presented as means ± S.D. ns: not significant, *p < 0.05, **p < 0.01. (*N* = 5).

The depth of invasion, a quantitative measure of cancer cell infiltration, was significantly greater in all 3D models containing HSC-3 cells than in those with HSC-2 cells, regardless of the TME or culture duration (Fig. 5A,B). Compared with the 3D cancer-NOF models, the 3D cancer-CAF models showed significantly deeper invasion across all conditions, independent of OSCC cell type or manufacturing period, with the exception of the 3D HSC-2 model at 14 days of culture (Fig. 5A). All 3D cancer models showed a significant increase in invasion depth with extended culture duration (Fig. 5B).

### Quantitative and longitudinal analyses of 3D-reconstructed OCT images and comparative assessment of cancer cell invasion across 3D cancer models using two parameters

**Fig. 6:**
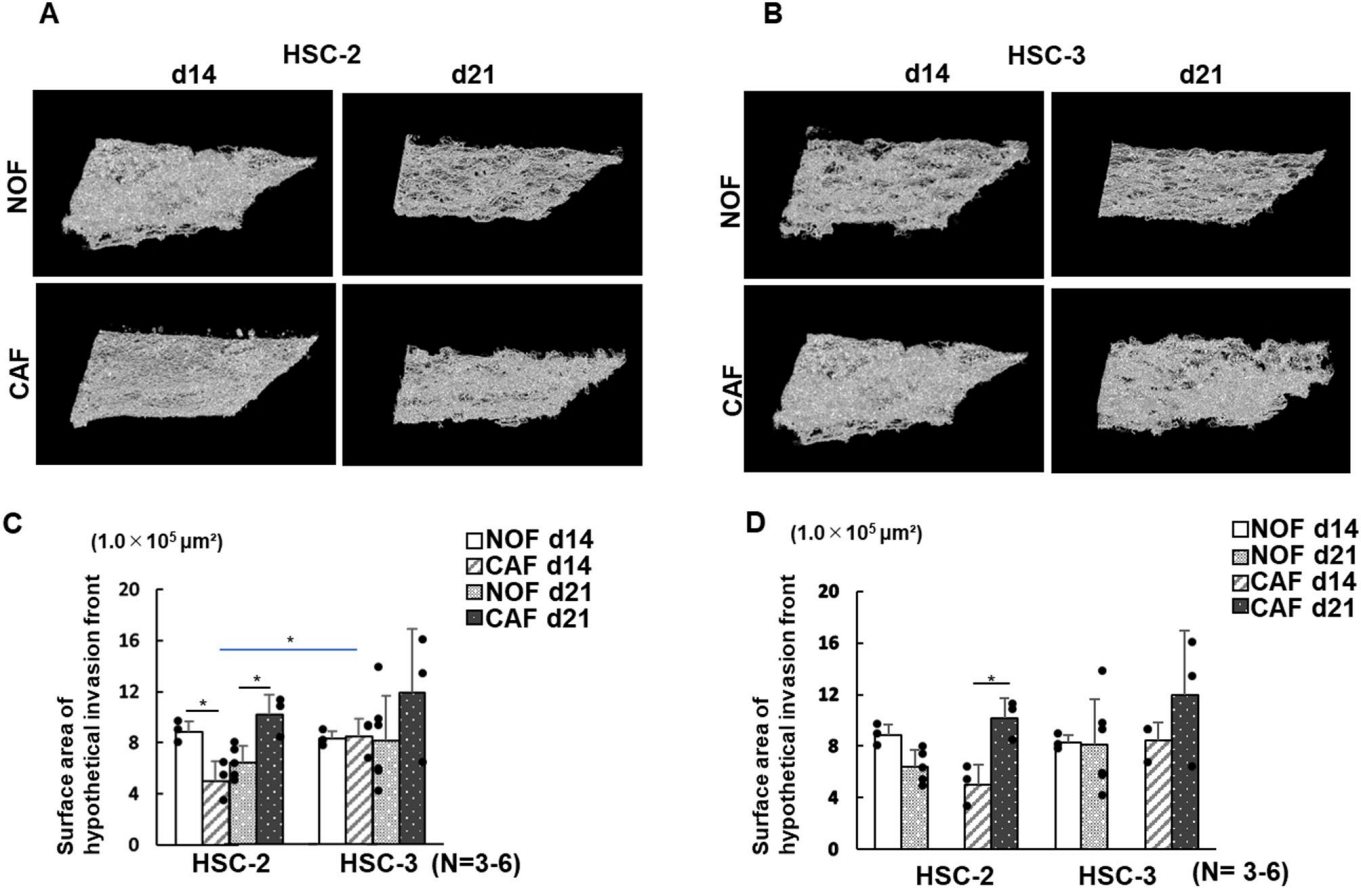
Representative surface images of the hypothetical invasive front reconstructed from OCT data (**A** and **B**) and planimetric measurements of surface area (**C**, **D**) in 3D oral cancer models. (**A**) Surface images of the hypothetical invasive front in HSC-2 cells under four different conditions. (**B**) Surface images of the hypothetical invasive front in HSC-3 cells under four different conditions. (**C**) Quantitative comparison of surface area across four conditions (HSC-2 vs. HSC-3 and CAFs vs. NOFs). Data are presented as means ± S.D. ns: not significant, *p < 0.05, **p < 0.01. (*N* = 3–6). (**D**) Longitudinal comparison of surface area in the same models over time (14 vs. 21 days). Data are presented as means ± S.D. ns: not significant, *p < 0.05, **p < 0.01. (*N* = 3–6).

**Fig. 7:**
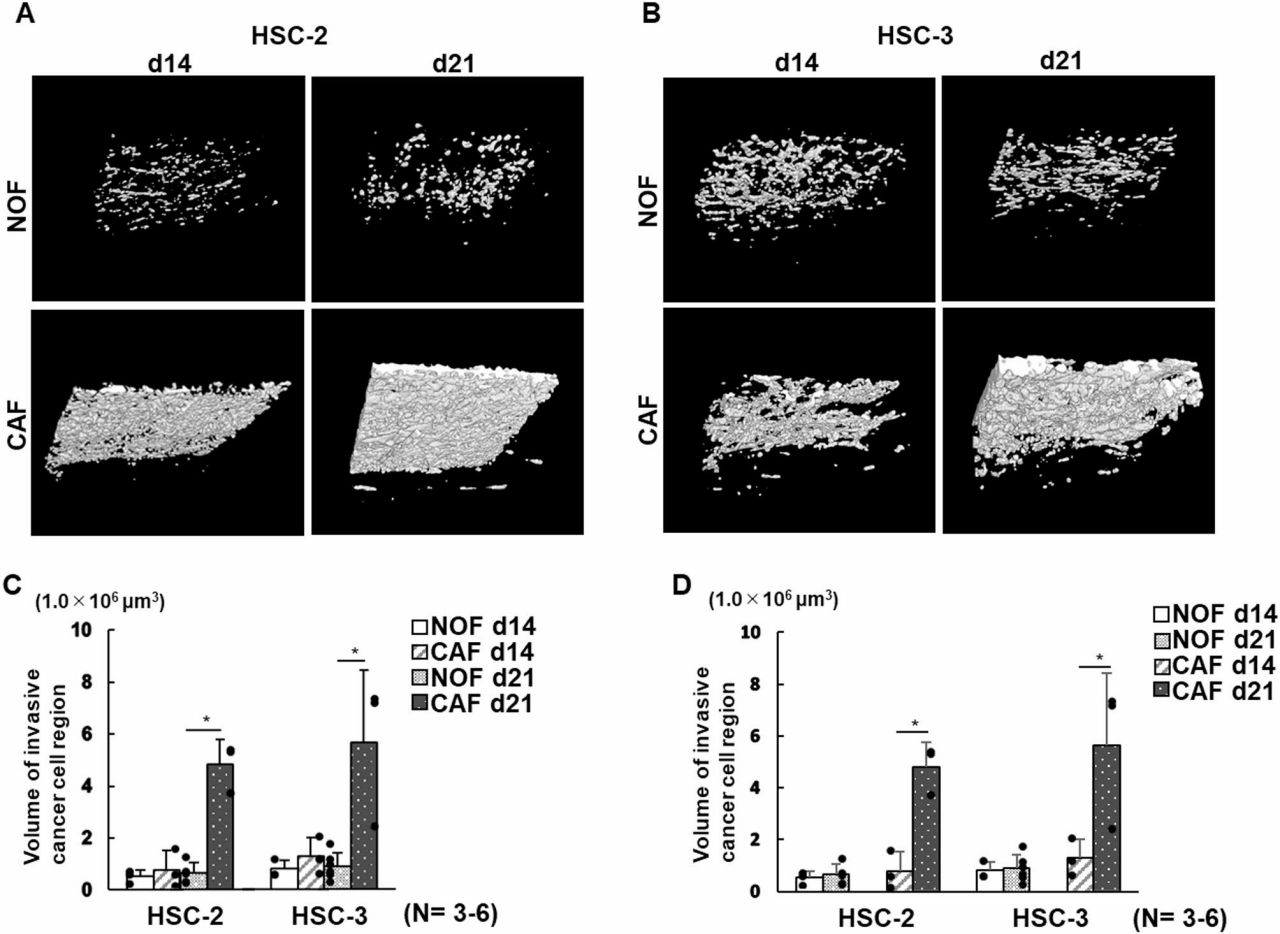
Representative 3D OCT reconstructions of the invasive cancer cell region (**A** and **B**) and volumetric measurements in 3D oral cancer models (**C** and **D**). (**A**) Three-dimensional reconstructions of invasive cancer cell regions in the HSC-2 model under four different conditions. (**B**) Three-dimensional reconstructions of invasive cancer cell regions in the HSC-3 model under four different conditions. (**C**) Quantitative comparison of total invasive cancer cell volume across four conditions (HSC-2 vs. HSC-3 and CAFs vs. NOFs). Data are presented as means ± S.D. ns: not significant, *p < 0.05, **p < 0.01. (*N* = 3–6). (**D**) Longitudinal comparison of total invasive cancer cell volume in the same model over time (14 vs. 21 days). Data are presented as means ± S.D. ns: not significant, *p < 0.05, **p < 0.01. (*N* = 3–6).

To monitor cancer cell invasion quantitatively and longitudinally, 3D images of the cancer models were reconstructed from OCT imaging using deep learning. This approach successfully enabled the measurement of two parameters, namely, the surface area of the HIF (Fig. 6A,B) and the total volume of the ICCR (Fig. 7A,B).

The HIF surfaces appeared convoluted, as expected within the ICCR in the 3D cancer models (Fig. 6A,B). Quantitative analysis revealed limited variation in surface area among the HSC-2 models, whereas the variation was larger in the HSC-3 CAF model cultured for 21 days. A notably larger surface area was observed only in the HSC-3 CAF model cultured for 21 days (Fig. 6C). Compared with the HSC-2 models, the surface area was significantly larger only in the HSC-3 CAF model cultured for 14 days (Fig. 6C). Significant differences were also observed between CAF and NOF models in the HSC-2 model, regardless of the culture duration (Fig. 6C). However, no significant differences were observed between the NOF and CAF models in the HSC-3 model (Fig. 6C). Longitudinal analysis showed that only the HSC-2 CAF model cultured for 21 days exhibited a significantly larger surface area than its NOF counterpart, whereas no time-dependent differences were observed in any of the other models (Fig. 6D).

The reconstructed 3D images of the ICCR were also visualized and fully separated from the original cancer cell region (Fig. 7A,B; Supplementary Movies 3 and 4). Quantitative analysis showed that the total volume of the ICCR was significantly larger in the HSC-2 and HSC-3 CAF models cultured for 21 days than in any of the other 3D cancer models (Fig. 7C). However, when comparing HSC-2 and HSC-3 models, no significant differences were observed. In the longitudinal analysis, the total ICCR volume was significantly greater in the CAF models cultured for 21 days than those cultured for 14 days. No significant differences were observed between any other model conditions, regardless of cancer cell type or culture duration (Fig. 7D). Despite these variations, the outcomes suggest that 3D images derived from OCT are a valuable tool for monitoring cancer cell invasion.

### Comparison between indexes of cancer cell invasion determined by histomorphometric analysis and 3D reconstructed OCT images

**Fig. 8:**
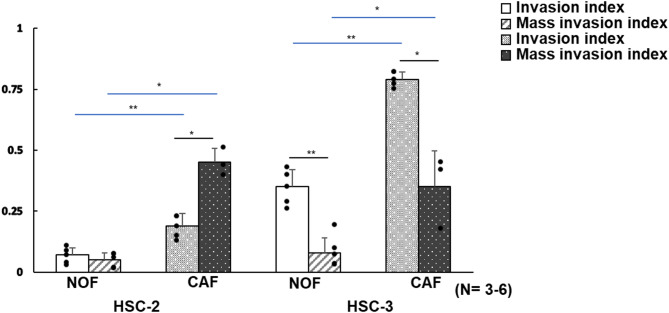
Comparison of mass invasion index from OCT-based volumetric analysis with invasion index from histomorphometric analysis in four 3D cancer models cultured for 21 days. Data are presented as means ± S.D. ns: not significant, *p < 0.05, **p < 0.01 (*N* = 3–6).

Understanding the quantitative correspondence between the invasion index and mass invasion index obtained from the same 3D cancer model at the endpoint is essential for validating the reliability of 3D reconstructed OCT images. To verify that the features identified in 3D OCT images align with histomorphometric parameters, we compared the trends of these indexes across four model types cultured for 21 days and examined their correlations. In HSC-2 models, only the CAF model showed a significantly higher mass invasion index (Fig. 8). In contrast, for HSC-3 cells, both NOF and CAF models showed remarkably lower mass invasion index values (Fig. 8). Additionally, the 3D cancer-CAF models exhibited a higher mass invasion index than the 3D cancer-NOF models, reflecting the trend observed in the invasion index derived from histomorphometric analysis for both HSC-2 and HSC-3 cells (Fig. 8). Although the absolute values differed between the invasion index and the mass invasion index, both indexes showed statistically significant results. These findings suggest that 3D images reconstructed from OCT correlate closely with histomorphometric parameters of cancer cell invasion.

## Discussion

SD-OCT enables high-resolution, real-time cross-sectional imaging of biological tissue microstructures, whereas Swept‑Source OCT offers greater penetration depth^30^. Consistent with our previous study using OCT to monitor tissue-engineered oral mucosa^22^, the present work successfully achieved structural visualization and regional differentiation within a 3D organotypic oral cancer model at high spatiotemporal resolution. To the best of our knowledge, this is the first study to use SD-OCT in combination with a deep learning model to 3D oral cancer models for the quantitative and longitudinal monitoring of cancer cell invasion, thereby confirming the applicability of OCT to our oral cancer model.

SD-OCT is effective for noninvasively evaluating cancer cell behavior and structural changes in 3D models such as spheroids and organoids^15,31,32^. In spheroids, 3D dynamic OCT enables label-free, longitudinal analysis of growth, cavitation, and drug responses, including necrotic core formation^31,33^. In organoid clusters, deep learning–assisted segmentation allows accurate tracking of organoids over 50 μm for assessing growth and structural heterogeneity^34^. However, our approach is unique in that it integrates SD-OCT with an organotypic culture model of OSCC, which more closely mimics the TME of oral cancer and replicates spatial interactions between cancer cells and CAFs. This application enables high-resolution, spatiotemporal monitoring of cancer progression, allowing visualization of 3D cancer cell invasion and interaction dynamics between cancer cells and fibroblasts in the stromal layer, without requiring paraffin embedding or staining. This novel integration of SD-OCT with an organotypic culture model is a powerful and versatile tool for visualizing tumor biology and evaluating drug responses in personalized therapies.

We were able to identify the bilayered structure of the 3D organotypic cancer model, in which the original cancer cell region exhibited strong signal intensity, whereas the underlying stromal layer showed weak signal, similar to cellular-resolution images of multilayered human skin *in vivo*^35^. Additionally, using a deep learning model trained to identify cancer cells based on differences in brightness within OCT images, we were able to depict infiltrating cancer cells separated from the original cancer cell region within the stromal layer. Because standard spheroids and organoids cannot replicate cancer invasion behavior, imaging the 3D organotypic cancer model represents a novel and innovative advancement. Although limited by penetration depth in thicker samples, SD‑OCT remains preferable for its high-resolution, cross-sectional imaging capabilities. Thus, OCT is a promising tool with increasing potential to assess treatment outcomes in future oral cancer studies.

Although 3D imaging reconstructed from histological sections has advanced our understanding of cancer cell invasion and TME, the reconstruction process is extremely labor-intensive^36–38^. Recent spatial phenotyping technologies enable innovative analyses of cancer cell invasion and TME in *in vivo* tissues within a short time. However, high costs, technical challenges, including limitations in 3D resolution and longitudinal tracking, and a lack of standardized protocols remain significant challenges^39^. SD-OCT imaging data provide quantitative monitoring of lesion progression or regression through signal attenuation, thickness measurements, and scattering profiles^8,40–43^. In our previous work, we integrated deep learning for automated 3D reconstruction from OCT images^22^. This study demonstrated that objective parameters obtained from reconstructed 3D OCT images, such as the surface area of the HIF and the total volume of the ICCR, can be effectively applied for simple screening and monitoring of cancer cell invasion. Comparisons between 3D cancer models comprising CAFs and NOFs further support the value of our system for TME research, particularly for longitudinal studies, as our organotypic oral cancer models closely recapitulate *in vivo* TME^17^.

Based on 3D OCT images obtained from the same 3D model, two cancer invasion parameters were designed to examine the feasibility of OCT-based 3D reconstructions. Although the HIF surface was well visualized and appeared convoluted, results varied among the eight 3D cancer model conditions. Consequently, the surface area of the HIF may be less specific for monitoring cancer cell invasion, possibly because of the exclusion of cancer cell clusters/islands detached from the original cancer cell region and inclusion of finger-like invasion only during calculation. In contrast, the total volume of ICCR showed significant differences between CAF and NOF models for both OSCC cell types on Day 21 as well as Days 14 and 21 for the CAF models. However, the time points of Days 14 and 21 for evaluating cancer invasion in this study were selected for convenience, based on our manufacturing protocol, which involved a substantial change in culture conditions every 7 days. OCT imaging can be used to evaluate the samples at any time point; therefore, cancer cell invasion dynamics in our 3D oral cancer models must be examined in more flexible time points such as shorter intervals or longer span. Nonetheless, these findings support the feasibility of reconstructed 3D OCT imaging for quantitative and longitudinal analysis of invasive cancer behavior, positioning this approach as a promising tool for future *in vitro* cancer cell invasion studies.

In the clinical setting of oral cancer, histopathological findings from surgical specimens used to indicate aggressiveness, metastatic potential, and postoperative treatment guidelines include the worst pattern of invasion (WPOI) and tumor budding, both of which correlate with patient survival^44–47^. In our 3D OCT images, the invasion patterns resemble WPOI types I-II, which are characterized by cohesive invasion rather than individual cells or small clusters infiltrating the deep stromal layer. Although the HSC-3 CAF model showed histological features similar to WPOI type III, the number of cells represented in the volumetric ICCR images remains unclear. When using OCT and deep learning–based volumetric analysis, small cancer islands (fewer than 15 cells) may not be reliably detected due to current resolution limitations. Therefore, the current resolution accuracy of OCT in detecting infiltrating small cancer clusters remains inferior. Further technological advancements are required to determine whether OCT can resolve cancer islands comprising ≤ 15 cells.

The mass invasion index was higher than the invasion index in the HSC-2 CAF models, despite tissue shrinkage caused by fixation artifacts. In contrast, the mass invasion indexes were lower than the invasion index in HSC-3 cells. This discrepancy could be attributed to the limited OCT resolution, which may miss small infiltrating cancer clusters. Furthermore, this parameter appears useful for evaluating cohesive invasion patterns. Because both the invasion index and mass invasion index exhibited significant differences while following the same increasing trend, their correlation suggests that 3D OCT image–based data are reliable and consistent with histomorphometric data.

In conclusion, label-free OCT imaging preserves sample integrity and enables quantitative, longitudinal monitoring of invasive behavior of cancer. These results confirm the applicability and feasibility of OCT imaging in our 3D cancer model. Thus, our novel approach of integrating 3D organotypic oral cancer models with OCT enables real-time monitoring of tumor development and treatment responses, providing a more relevant alternative to traditional 2D cultures. This technique holds promise for advancing personalized medicine by facilitating drug screening and biomarker discovery in clinically relevant models. Therefore, SD-OCT may emerge as a novel imaging tool in cancer research and cutting-edge biomedical studies based on 3D cultures.

## Supplementary Information

Below is the link to the electronic supplementary material.

Supplementary Material 1

## Data Availability

The data underlying this article are available from the corresponding author on reasonable request.

## Abbreviations

CAFs: Cancer-associated fibroblasts
NOFs: Normal oral fibroblasts
OCT: Optical coherence tomography
OSCC: Oral squamous cell carcinoma
3D: Three-dimensional
TME: Tumor microenvironment
