# Supplementary figures and images for: Quantitative and longitudinal monitoring of cancer cell invasion in a three-dimensional in vitro model of oral cancer using optical coherence tomography
Source: Sci Rep. 2025 Nov 27;15:45449. doi: 10.1038/s41598-025-28471-y (PMC12749817; doi:10.1038/s41598-025-28471-y)

## Slide 1
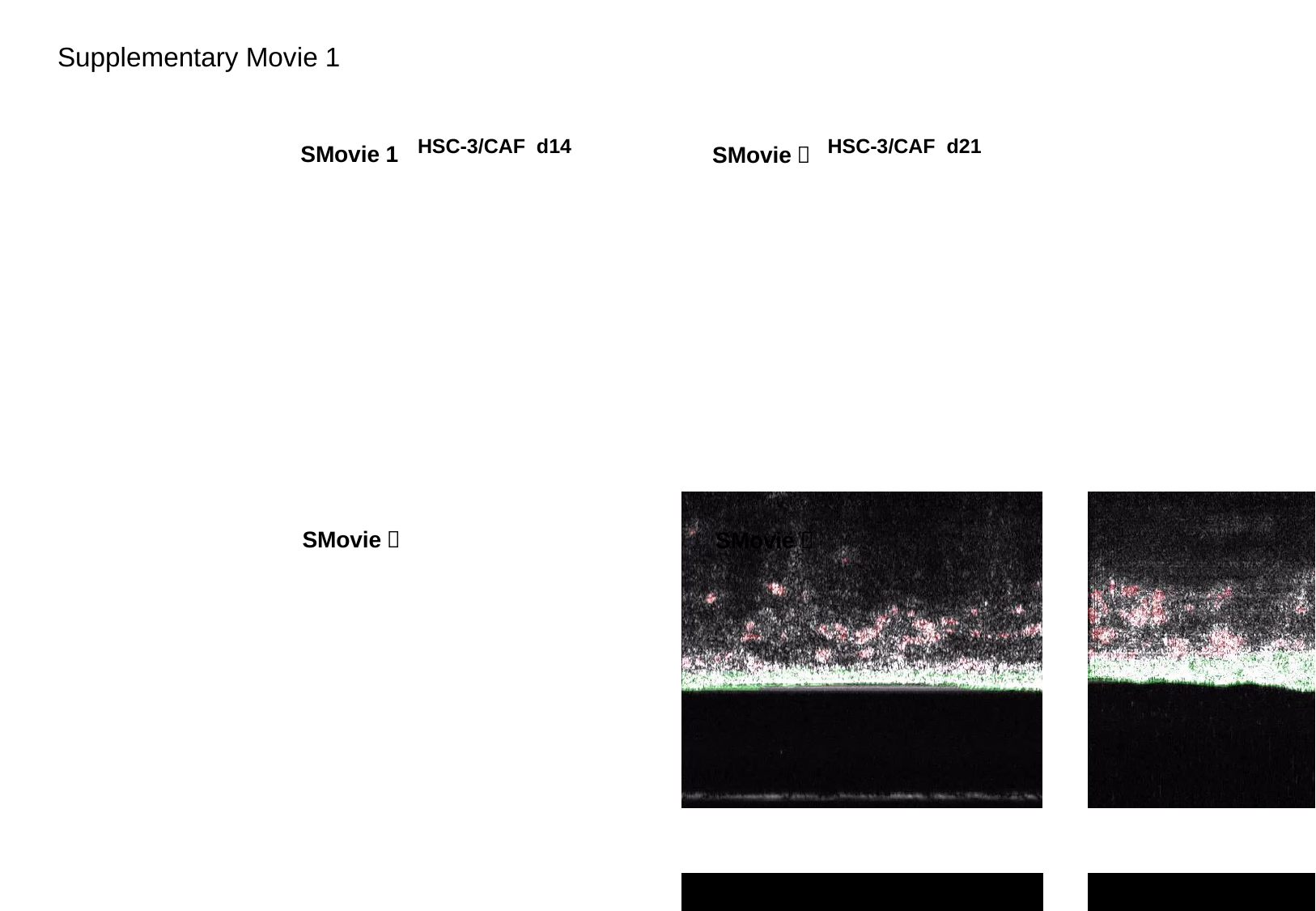

Supplementary Movie 1
HSC-3/CAF d14
HSC-3/CAF d21
SMovie 1
SMovie２
SMovie３
SMovie４

Supplement: Supplementary file 1 — Supplementary Material 1 [file 41598_2025_28471_MOESM1_ESM.pptx]
